# The personal and professional impact of COVID-19 on orthopedic surgery trainees: reflections from an incoming intern, current intern, and chief resident

**DOI:** 10.1080/17453674.2020.1789310

**Published:** 2020-07-03

**Authors:** David N Bernstein, Nattaly Greene, Ishaq O Ibrahim

**Affiliations:** Harvard Combined Orthopaedic Residency Program, Massachusetts General Hospital, Boston, USA

On December 31, 2019, Chinese health officials reported to the World Health Organization (WHO) Country Office (World Health Organization 2020) growing concern regarding an increasing number of cases of pneumonia of unknown cause. Since that time, numerous public health interventions have been introduced (Patel and Jernigan 2020), a global pandemic was declared (Cucinotta and Vanelli 2020), and much was learned about the novel coronavirus (i.e., SARS-CoV-2), the microscopic culprit behind the world’s ongoing public health and economic crises. In addition, the COVID-19 global pandemic has had a notable impact on health systems globally, placing a strain on resources and reducing or halting non-emergent medical and operative care.

Much has been written about COVID-19 and its impact on orthopedic surgery (Jain et al. 2020, Ranuccio et al. 2020), including on orthopedic surgery trainee education (Kogan et al. 2020, Schwartz et al. 2020, Stambough et al. 2020). Further, there is a growing focus and commentary on physician well-being—both physical and mental (Massey et al. 2020). However, transparent COVID-19 perspectives by orthopedic surgery trainees—on personal and professional levels—have been limited. While the delivery of high-quality orthopedic care and education remains central to the musculoskeletal health of patients globally, we must also pause for reflection, a crucial element of narrative medicine that has a number of benefits for patient care and provider well-being (Charon 2001).

In this perspective, we share the reflections of 3 orthopedic surgery trainees at a single academic training program in a large city, Boston, in the United States: 1 incoming intern; 1 current intern; and 1 chief resident. While all are trainees, the difference in training year is crucial to illuminate the different professional stressors. Additionally, each individual has their own personal stressors and reactions to the ongoing crisis. We conclude this perspective by summarizing key themes and urging our orthopedic surgery trainee colleagues globally to engage in a similar exercise.

## Professional reflections

### Chief resident (IOI)

As a burgeoning surgeon in my final year of residency, the pandemic has appeared to strike at a particularly inopportune time. In many ways, I viewed my last year of residency as my “final act” as a trainee prior to moving on to the increased responsibility and autonomy of fellowship and, subsequently, my attending career. Now several weeks into the pandemic, personal excitement for a strong finish has been replaced by unease and discontentment.

When social distancing policies began to take hold in March, I was settling into my role as service chief for 1 of our busy arthroplasty services. The high-volume rotation would have afforded me plenty of additional “reps” to maximize my proficiency in arthroplasties prior to moving on to trauma fellowship in the summer. The COVID-19 pandemic swiftly brought that opportunity to an end. The lost training and uncertainty regarding our return to familiar affairs has left me with an odd sense of unfulfillment as I prepare to take the next steps in my career.

At the clinical level, COVID-19 has entirely altered our workflow. As our resident pool is thinned by redeployments to the emergency department (ED) and surgical intensive care unit (SICU), coverage of the orthopedic trauma service and other emergent or urgent cases has become a collective effort amongst the remaining residents. Persistent limitations of COVID-19 testing and ever-evolving perioperative protocols secondary to the virus have been frequent sources of frustration. Additionally, loss of the dedicated “trauma room” has made the workday less predictable.

Overall, support from our program leadership has been thoughtful, compassionate, and frequent. As an example, our residency program director and hospital department chairs updated residents on a daily basis. One of the core goals of this communication was to keep us abreast of important hospital trends and the possible implications for us as trainees (e.g., redeployment to another service/area of the hospital or schedule restructuring). This daily insight was critical in maintaining camaraderie and morale in an unprecedented time.

### Current intern (NG)

COVID-19 was deemed to be a global pandemic 2 weeks prior to the start of our intern bootcamp month—a month dedicated to teaching first-year residents critical orthopedic knowledge and skills. At the time, I was on a general surgery rotation at 1 of our community hospitals, and I witnessed notable changes daily as we prepared to face the crisis. Elective cases began dropping off our operating room (OR) boards, emails began flooding our inboxes, and schedules were rearranged in attempt to decrease the number of people who might be exposed to the virus. Our general surgery team, which typically includes 2 interns, a postgraduate year (PGY)-2, PGY-3, and a PGY-5, was distilled to me and the PGY-5.

As time progressed, it became clear that a virtual orthopedic surgery intern bootcamp would take the place of the traditional in-person month of orthopedic learning and co-intern bonding. To make this transition successful, a box of orthopedic surgery instruments was delivered to my home and a calendar of remote lectures to be delivered via web conference was created. Instead of developing my knowledge and skills alongside my co-interns in person, I have been glued to my computer screen, learning orthopedic surgery from afar. In addition, the hands-on portion of skill development has led to my small studio apartment being filled with a Black & Decker work bench, sawbones, drills, plates, sutures, and other essential instruments and equipment required to learn the basics of orthopedic surgery. Overall, I feel that I have been able to adapt and dedicate time to learning and developing my orthopedic surgery skills remotely.

My professional growth via a virtual bootcamp was only made possible because of the incredible support of program leadership, faculty, and the tireless work of our senior trainees. Without their remarkable effort to adapt teaching and learning opportunities to online platforms, I would not have had such a transformative learning experience.

### Incoming intern (DNB)

The final year of medical school in the United States is a rollercoaster ride. While the stress of visiting clinical clerkships and applying to residency begins the year, celebrations and extended travel typically conclude the year. Most medical students long for the final stretch of medical school, a sign that their years of hard work and dedication has paid off.

However, 2020 is not a typical year. As the global pandemic raged on, it became clear by early March that these traditional celebrations, including Match Day (i.e., the day when medical students who apply to complete their medical training in the United States learn where they will attend residency) and graduation, were not going to be held in person. In lieu of the pomp and circumstance, short virtual “celebrations” were planned and carried out. Despite the efforts and hard work of medical school leaders, I felt, and continue to feel, a gaping hole in our medical school experience—a lack of closure. Yet, I still feel a sense of professional pride and accomplishment, especially having matched into my desired field. Once residency begins, I firmly believe I will experience closure to my medical school journey.

Overall, the ongoing global pandemic has caused a notable disruption of the “normal” conclusion of medical school. Fortunately, however, even a global pandemic could not take away my remarkable mentors and their dedication to my professional growth; indeed, they have spent innumerable hours engaging with me via email and virtual meetings. Further, the clear messaging by my incoming orthopedic surgery residency program’s leadership has eased my anxiety and prepared me to enter the orthopedic surgery workforce during this unusual time in history.

## Personal reflections

### Chief resident

During the COVID-19 pandemic, concern over the well-being of my family, friends, and peers has been a constant source of stress. Increased downtime, however, has allowed me time to frequently connect with my family and even reconnect with friends from earlier stages of my life. During more “normal” times, this might not have been possible. Additionally, closure of “nonessential” services, including the gym and barbershop, has forced us to be resourceful and explore new ways to exercise, cook, and groom at home. These endeavors have been mostly successful and have helped us to maintain our sanity during these difficult times.

### Current intern

Throughout this crisis, I have felt more anxious and preoccupied; however, these feelings have often been mixed with a sense of overwhelming gratitude for my health and for the health of my family. My core concerns have revolved around my family members, including their health and their financial well-being. As the pandemic evolved, it became clear that its impact started to affect communities of color disproportionally. Thus, additional concerns included trying to understand the root cause of this inequity and how I could best help my community. I believe that the best antidote for my anxiety has been going into work and feeling useful. We went into medicine with the intention of helping others, and as orthopedic surgeons we find particular joy in fixing problems. Therefore, being at the hospital, doing meaningful work, and caring for others has been energizing.

Additionally, I have developed a workout routine, which has been an important way for me to look after myself. Reincorporating exercise has been very therapeutic, and it has helped to break up each day. Also, I have resumed journaling and writing to friends and family as a way to reflect on this unique experience. Lastly, I have noticed that I am frequently using the FaceTime application on my iPhone to chat face-to-face with family and friends. I have reconnected as well with those with whom I had previously lost contact. This has helped me fight the feeling of isolation.

### Incoming intern

Throughout the COVID-19 global pandemic, I have had a persistent level of uneasiness—a pit in my stomach. While many of my colleagues across the country graduated medical school early to join the frontlines, my training program did not require me to begin residency early. At times, I felt a sense of guilt for not being directly engaged in patient care, especially because I felt as if I could help—even if that meant simply handing out masks or organizing documents.

In addition to my own uneasiness, I had a heightened level of angst surrounding the health of my family. During the pandemic, I have been fortunate to spend time in person with my family. However, my father, a pediatrician, has continued to deliver in-person care to children. Further, my parents are in an age range that increases their risk of COVID-19 complications, and my sister is pregnant. Despite each member of my family taking all the necessary safety precautions, there remains a possibility of infection in 1 or more of them. However, because my family has remained healthy to date, I have had a constant sense of gratitude, especially as I witness so many around the world suffering. Further, I am thankful that I was able to utilize the “Stay At Home” order to spend high-quality time with those who matter most to me.

What one word would you use to describe your feelings about the current COVID-19 global pandemic?

Chief resident: Ambiguity.

Current intern: Uncertainty.

Incoming intern: Grit.

When I look back on this public health crisis in 10 years, I will remember…

Chief resident: … the leadership, strength, and resilience of our healthcare communities.

Current intern: … my work with the Latinx community as part of the Spanish Language Care Group at our tertiary care hospital. I will never forget the sense of relief that I witnessed on patients’ faces or heard in patients’ voices as they first saw me or heard me over the phone communicating in their native language. Additionally, I will always remember how rewarding and gratifying it was for me to be able to contribute in a meaningful way and to ensure that Spanish-speaking patients had a voice during this crisis.

Incoming intern: … the stories of inspirational everyday heroism, which reinforced my passion for helping others through a career in orthopedic surgery.

## Final thoughts

Three key themes transcend our individual reflections. A common theme shared by all of us is a heightened sense of anxiety. While similar reasons underlie this feeling from a personal standpoint (i.e., concern about the well-being of family and friends), there are differences in the cause of uneasiness from a professional standpoint. This is likely secondary to the different levels of orthopedic surgery training we have each completed to this point in time. Another theme in our responses focused on family, friends, and community. Each of us was, and continues to be, most worried not about ourselves but about those around us. From a community standpoint, COVID-19 magnified the presence of systemic issues in our society, including race-related inequity and healthcare disparities such as burden of illness and death (US Centers for Disease Control and Prevention 2020). For example, many in minority communities often hold essential jobs that do not allow for them to work from home. Unfortunately, there is no easy fix to this inequity; however, discussion, education, and pledging to take action is crucial to make substantive change. Further, we took the additional time available to us, given the pandemic, to connect with family and friends more consistently. Another consistent theme is the deep appreciation of support and leadership in a challenging time. Indeed, despite the uncertainties around COVID-19, we each appreciated the dedication of faculty; they have sacrificed much to ensure our continued professional development and personal well-being. Further, leadership provided and encouraged communication, making it easy and acceptable to communicate with them regarding personal matters and struggles, as well as seek out resources for help, if needed.

By reflecting on COVID-19 and its impact on each of us—both professionally and personally—we hope to normalize many of the feelings experienced by other orthopedic surgery trainees. We urge our fellow trainees in the United States and around the globe to share their reflections and insights on their own experiences as well. This can be done via social media, peer-reviewed journal articles, or other means. Not only may “best practices” be able to be appreciated, but the healing process can begin. The future of health care and musculoskeletal care is bright. If all of us in orthopedic surgery—from attending surgeons to trainees—fully support one another, we will move beyond the COVID-19 global pandemic physically and mentally stronger than ever.

## Funding, potential conflicts of interest

No funding was received in support of this perspective. All authors (DNB, NG, IOI) declare no related potential conflicts of interest. 
